# Sex-Specific Differences in Antidepressant and Antipsychotic Treatment Outcomes and Serum Levels in Children and Adolescents

**DOI:** 10.3390/pharmaceutics17080983

**Published:** 2025-07-30

**Authors:** Maike Scherf-Clavel, Stefanie Fekete, Manfred Gerlach, Christoph U. Correll, Paul Plener, Jörg M. Fegert, Andreas Karwautz, Peter Heuschmann, Tobias Banaschewski, Wolfgang Briegel, Christian Fleischhaker, Tobias Hellenschmidt, Hartmut Imgart, Michael Kaess, Michael Kölch, Karl Reitzle, Tobias J. Renner, Christian Rexroth, Gerd Schulte-Körne, Frank Theisen, Susanne Walitza, Christoph Wewetzer, Franca Keicher, Stefan Unterecker, Sebastian Walther, Marcel Romanos, Karin M. Egberts, Timo Vloet, Regina Taurines

**Affiliations:** 1 Department of Psychiatry, Psychosomatics and Psychotherapy, Center of Mental Health, University Hospital of Wuerzburg, 97080 Wuerzburg, Germany; 2 Department of Child and Adolescent Psychiatry, Psychosomatics and Psychotherapy, Center of Mental Health, University Hospital Wuerzburg, 97080 Wuerzburg, Germany; 3 Department of Child and Adolescent Psychiatry, Charité Universitätsmedizin Berlin, 13353 Berlin, Germany; 4 The Zucker Hillside Hospital, Department of Psychiatry, Northwell Health, Glen Oaks, NY 11004, USA; 5 Donald and Barbara Zucker School of Medicine at Hofstra/Northwell, Department of Psychiatry and Molecular Medicine, Hempstead, NY 11549, USA; 6 Department of Child and Adolescent Psychiatry/Psychotherapy, University Hospital Ulm, 89075 Ulm, Germany; 7 Department of Child and Adolescent Psychiatry, Medical University Vienna, 1090 Vienna, Austria; 8 Institute of Clinical Epidemiology and Biometry, University of Wuerzburg, 97080 Würzburg, Germany; 9 Department of Child and Adolescent Psychiatry and Psychotherapy, Central Institute of Mental Health, Medical Faculty Mannheim, Heidelberg University, Partner Site Mannheim-Heidelberg-Ulm, 68159 Mannheim, Germany; 10 Department of Child and Adolescent Psychiatry, Psychosomatics and Psychotherapy, Leopoldina Hospital, 97422 Schweinfurt, Germany; 11 Department of Child and Adolescent Psychiatry and Psychotherapy, University Medical Center Freiburg, 79104 Freiburg, Germany; 12 Department for Child and Adolescent Psychiatry, Psychotherapy and Psychosomatic Medicine, Vivantes Clinic Berlin Neukölln, 12351 Berlin, Germany; 13 Parkland-Clinic, Clinic for Psychosomatics and Psychotherapy, Academic Teaching Hospital for the University Gießen, 34537 Bad Wildungen, Germany; 14 Clinic for Child and Adolescent Psychiatry, Center for Psychosocial Medicine, University Hospital Heidelberg, 69115 Heidelberg, Germany; 15 Department of Child and Adolescent Psychiatry and Psychotherapy, University Hospital for Child and Adolescent Psychiatry and Psychotherapy, University of Bern, 3000 Bern, Switzerland; 16 Department of Child and Adolescent Psychiatry and Psychotherapy, Brandenburg Medical School Brandenburg, 16816 Neuruppin, Germany; 17 Department of Child and Adolescent Psychiatry, Neurology, Psychosomatics, and Psychotherapy, University Medical Center Rostock, 18147 Rostock, Germany; 18 Specialist Practice and Medical Care Center for Child and Adolescent Psychiatry Munich, 81241 Munich, Germany; 19 Department of Child and Adolescent Psychiatry, Psychosomatics and Psychotherapy, Center of Mental Health Tuebingen, University Hospital of Psychiatry and Psychotherapy Tuebingen, 72076 Tuebingen, Germany; 20 Clinic for Child and Adolescent Psychiatry, Psychosomatics and Psychotherapy at the Regensburg District Hospital, Medbo KU, University Hospital Regensburg, 93053 Regensburg, Germany; 21 Department of Child and Adolescent Psychiatry, Psychosomatics and Psychotherapy, Ludwig-Maximilians-University (LMU) Hospital, 80097 Munich, Germany; 22 Herz-Jesu-Krankenhaus gGmbH, Department of Child and Adolescent Psychiatry and Psychotherapy, 36037 Fulda, Germany; 23 Department of Child and Adolescent Psychiatry and Psychotherapy, University Hospital of Psychiatry Zurich, 8032 Zurich, Switzerland; 24 Kliniken der Stadt Köln gGmbH, Clinic for Child and Adolescent Psychiatry Holweide, Children’s Hospital Amsterdamer Straße, 50825 Cologne, Germany; 25 Department of Psychiatry and Psychotherapy, Social Foundation Bamberg/Michelsberg Clinic, 96047 Bamberg, Germany; 26 Department of Child and Adolescent Psychiatry, GGZ Reinier van Arkel, 5211 LJ ‘s-Hertogenbosch, The Netherlands

**Keywords:** pharmacodynamics, therapeutic drug monitoring, psychotropic drugs, adverse drug effects

## Abstract

**Introduction:** Sex-specific differences in psychopharmacological treatment have gained increasing attention in adults, with studies showing that women often have higher serum concentrations of psychotropic drugs due to biological differences. However, despite recognition of these differences in adults, reference ranges for therapeutic drug monitoring (TDM) in general, but even more sex-specific therapeutic windows for psychotropic drugs, are lacking in children and adolescents, who may metabolize and respond to medications differently. **Aim:** The study aimed to investigate sex-specific differences in antidepressant (AD) and antipsychotic (AP) -treatment outcomes, and pharmacokinetics in childhood/adolescence. In particular, we examined differences in AD and AP serum levels and clinical effects, including adverse drug effects (ADEs) and therapeutic effectiveness. **Methods:** This study is part of the multicenter “TDM-VIGIL” pharmacovigilance project, which prospectively followed patients aged 6–18 years treated with AD and AP across 18 child psychiatric centers in German-speaking countries from 2014 to 2018. Clinical data, including drug concentrations (AD: fluoxetine, mirtazapine, (es)citalopram, sertraline; AP: aripiprazole, quetiapine, olanzapine, risperidone), were collected using an internet-based registry, and treatment outcomes and ADEs were assessed during routine visits. Statistical analyses were performed to examine sex differences in pharmacokinetics and clinical responses, adjusting for age, weight, and other confounders. **Results:** A total of 705 patients (66.5% girls, 24.7% <14 years, mean age of 14.6 years) were included. Female patients were slightly older, had lower body weight, and were more often diagnosed with depression and anorexia nervosa, while boys were more frequently diagnosed with hyperkinetic disorders and atypical autism. We found no sex differences in the serum concentrations of investigated drugs when adjusted for age and weight. In fluoxetine treatment in patients diagnosed with mood (affective) disorders, female sex was associated with the probability for very good therapy response (*p* = 0.04), as well as with moderate treatment response (*p* = 0.02) compared to no treatment response. **Discussion**: Our findings suggest that sex may not affect serum levels of investigated AD and AP in children/adolescents. However, treatment outcome of fluoxetine was associated with sex, with higher probability for a better outcome in female patients diagnosed with mood (affective) disorders.

## 1. Introduction

In psychiatry, the consideration of sex-specific differences in psychopharmacological treatment has become increasingly important.

In adults, the sex differences in drug pharmacokinetics are influenced by variations in body fat and water distribution, protein binding capacity, and excretion rates, as well as immunological and hormonal factors. These differences can in turn affect the efficacy and tolerability of psychotropic drugs [1,2]. Numerous Therapeutic Drug Monitoring (TDM) studies on adults showed that women generally have 20–33.5% higher serum levels of psychotropic drugs [3,4]. This has been shown, for example, for the antipsychotics (AP) risperidone [3,5], clozapine [3], olanzapine [3,4], and quetiapine [3], as well as the antidepressants (AD) venlafaxine [5,6], amitriptyline [7], citalopram [7], doxepin [7], and mirtazapine [7].

TDM is a valuable tool for measuring drug levels of psychotropic drugs, which also allows to account for influencing factors such as sex. In practice, TDM is used to monitor the individual drug levels of psychotropic drugs and to titrate them into the recommended therapeutic reference range [8]. For the age group of minors, there is a general indication to apply TDM [8].

Children differ from adults not only in size, but in multiple pharmacokinetic parameters; therefore, some psychotropic drugs must be administered in disproportionately high doses compared to adults, as the child’s organism is able to metabolize and excrete certain substances more effectively in some cases [9]. AD and AP are typically used in child and adolescent psychiatry from school age (6–17 years). A large proportion of psychotropic drug prescriptions, 50% of AD and over 80% of AP, are prescribed ‘off-label’ [10]. Despite pharmacokinetic differences between children and adults, to date, defined therapeutic reference ranges for psychotropic drugs exist only for adults [8], and pharmacokinetic studies of psychotropic drugs in children and adolescents remain limited.

Previous pharmacokinetic studies on AD and AP in youth in small samples yielded equivocal findings regarding sex differences. For example, in children and adolescents treated with the AD fluoxetine, fluoxetine drug levels were higher in girls [11,12]. In contrast, other studies found no sex-specific differences in fluoxetine levels [13]. In AP, inconsistent findings were also reported. In clozapine, a higher mean serum concentration was found in girls [14] and no sex difference for the AP were found for risperidone [5,15,16], olanzapine [17], and tiapride [18]. However, it should be noted that the number of participants in these studies was small. In AP, a literature review on the impact of covariates like sex on the clinical pharmacokinetics in children and adolescents concluded that the topic has been poorly investigated for most AP and that findings were heterogeneous [19].

Thus, potential sex differences and their biographical timing require further investigation.

Serum levels may influence the effect of a psychotropic drug. Female sex is generally regarded as a risk factor for the occurrence of adverse drug effects (ADEs). Women show higher rates of ADEs during AD or AP treatment [20,21,22,23]; at the same time, AD and AP may also be more effective for females than for men [1,22]. A recent study with over 900 participants with depressive disorders, including children from the age of 12, showed that girls and women benefited more from AD medication than boys and men [24]. However, sex differences in pharmacodynamics in children and adolescents are also unknown.

Therefore, the aim of this study is to investigate whether there are sex-specific differences in the pharmacokinetics of AD and AP, as well as in AD and AP treatment outcomes and ADEs in childhood/adolescence. We hypothesized that, in line with adults, serum concentrations in female youth may be higher compared to male youth and that female sex may be a risk factor for ADEs. This naturalistic study contributes important insights into the optimization of psychopharmacotherapy in children and adolescents, with potential implications for both safety and efficacy. In addition, the study takes into account the frequent use of combination therapies in routine clinical practice, which is often excluded in controlled clinical trials but highly relevant for everyday psychiatric treatment in youth. Finally, the multicenter approach with centralized blood concentration measurements and transdiagnostic illness severity assessment via use of the CGI further enhance the value of this study.

## 2. Methods

### 2.1. Setting and Participants

Within the multicenter pharmacovigilance study ‘TDM-VIGIL’, funded by the federal Institute of Drugs and Medical Devices (BfArM-number V-15322/68605/2013-2018), patients aged 6–18 years treated with AD or AP for different mental disorders were prospectively followed from 2014 to 2018 in 18 child and adolescent psychiatric centers in three German-speaking countries. Diagnoses were assigned according to International Classification of Diseases 10th Revision (ICD-10) criteria by the treating child and adolescent psychiatrists following clinical examination and review of case files. Clinical as well as medication data were systematically documented using an internet-based patient registry [10]. Patients were divided according to their age into children (<14 years), and adolescents (≥14 years).

Written informed consent was obtained from each participant and the legal guardians. The project was authorized by the ethics committee of the lead study center (University Hospital Wuerzburg; 301/13_ff) as well as the local ethics committees of the participating centers. It is registered in the European Clinical Trials Database (EudraCT: 2013–004881-33), and was conducted according to the Declaration of Helsinki. For an overview of the study see Egberts et al., 2022 [10] and previous drug-specific analyses [25,26]. The only exclusion criteria in the study were (1) an absolute contraindication to the drug, and (2) concurrent participation in another clinical trial.

Pharmacological treatment, response assessment, blood collection, and laboratory drug concentration measurements were conducted within a routine healthcare setting via three different modalities, i.e., inpatient, outpatient, and day-treatment units. Experienced child and adolescent psychiatrists monitored the treatment course.

### 2.2. Study Medication

AD and AP were prescribed upon clinician’s choice either on- or off-label based on clinical judgment. The study protocol had no influence on the selection of the active substance, formulation, dosing regimen, and frequency, nor the duration of use. Both, combination therapy with different AD or AP, as well as simultaneous treatment with other psychotropic (e.g., psychostimulants, benzodiazepines, mood-stabilizers) or somatic drugs, were permitted and prescription details were recorded in the medication log.

### 2.3. Therapeutic Drug Monitoring and Pharmacokinetic Analyses

TDM was conducted according to the consensus guidelines of the Working Group on Neuropsychopharmacology and Pharmacopsychiatry (AGNP) [8]. Steady-state was assumed based on standard clinical practice. Patient adherence was assessed through routine clinical evaluation and by comparing recorded dosing information with measured serum levels. Importantly, the majority of patients were treated in inpatient settings, where medication intake is typically supervised, which further supports the assumption of medication adherence. All serum concentrations were determined at a steady-state trough level centrally at the laboratory for therapeutic drug monitoring of the university hospital of Wuerzburg, Germany. The concentrations of psychotropic drugs were determined using an isocratic reversed-phase high-performance liquid chromatography (RP-HPLC) system and a variable ultraviolet detector as described in detail elsewhere [27]. Dose-corrected serum concentrations (CD = serum concentration/dose) and metabolite-to-parent compound ratios (MPR = serum concentration metabolite/serum concentration parent compound) were calculated.

### 2.4. Assessment of Patient’s Characteristics, Clinical Response, and ADEs

All patients underwent a medical examination by a physician, including assessment of vital signs, electrocardiography, and laboratory blood tests. Clinical diagnoses were made by child and adolescent psychiatrists according to the ICD-10, chapter F.

The study included five main visits (baseline, visit at the target dose in steady state of the study medication, visit at discharge/end of outpatient treatment, follow-up visit two weeks after discharge (FU2-weeks), and follow-up visit 6 months (FU6-months) after the last steady-state visit) plus a non-binding number of additional visits. At each visit a standardized assessment of clinical response, ADEs and drug serum concentration (except FU2-weeks), was performed. Raters were trained experienced clinicians who were also blind to the serum concentrations at each visit.

To document the severity of the patients’ psychopathology and the change therein, the Clinical Global Impression subscales for severity and for improvement (CGI-S and CGI-I) [28] were used. The assessment of whether ADEs were present was carried out with Net-CGI during each visit.

### 2.5. Statistical Analyses

Statistical analyses were conducted in R v4.0.4 [29].

Comparing demographics and clinical characteristics of boys and girls in the sample, Mann–Whitney U tests and chi^2^ tests, or Fishers exact tests, respectively, were used. To expand specific analyses, linear regression analyses, or multinomial logistic regression analyses were conducted, including possible confounders (interaction sex*age (children vs. adolescents)). Children were defined as younger than 14 years, whereas adolescents were defined as 14 years and older. Differences in treatment outcome, and ADE between boys and girls were corrected for the interaction sex*age (children vs. adolescents), weight of the patients, as well as the number of administered drugs.

In pharmacokinetic—drug specific—analyses, differences between male and female patients regarding the serum concentrations were investigated using Mann–Whitney U tests. The dose-corrected serum concentration (CD) is the drug concentration in the blood divided by the administered dose, allowing comparison of drug exposure across different dosing levels. Sex-specific differences in CD of the drugs, as well as the metabolite-to-parent ratio (MPR) (if applicable) were investigated using linear regression analysis corrected for the interaction between sex*age (children vs. adolescents) of the patients, weight, and, if relevant, CYP-affecting comedication (CYP2D6-Inhibitors: fluoxetine, melperone, clomipramine, doxepin; CYP1A2-Inhibitor: fluvoxamine; CYP2C19-Inhibitor: fluoxetine, fluvoxamine [30]).

Drug-specific pharmacodynamic analyses focused on treatment outcome and ADEs and were conducted for schizophrenia, schizotypal and delusional disorders (F2x), mood (affective) disorders (F3x), and neurotic, anxiety, stress-related and somatoform disorders (F4x) diagnosed separately to overcome a bias due to the diagnosis. Multinomial logistic regression analysis was used to test for differences in outcome and in ADEs between male and female patients—corrected for the interaction sex*age (children vs. adolescents)—as well as the serum concentrations of the drug, weight of the patients, and the number of administered drugs. A *p*-value < 0.05 was considered significant. As this was an exploratory investigation, we did not correct for multiple testing.

## 3. Results

### 3.1. Demographic and Clinical Characteristics

In TDM-Vigil a total of 710 patients were included, 705 of whom received psychotropic medication. The mean age of the 705 patients (66.5% girls, 24,7% children < 14 years) was 14.6 years (SD 2.2, range 6–18). Demographic and clinical characteristics are summarized in Table 1.

**Figure 1:**
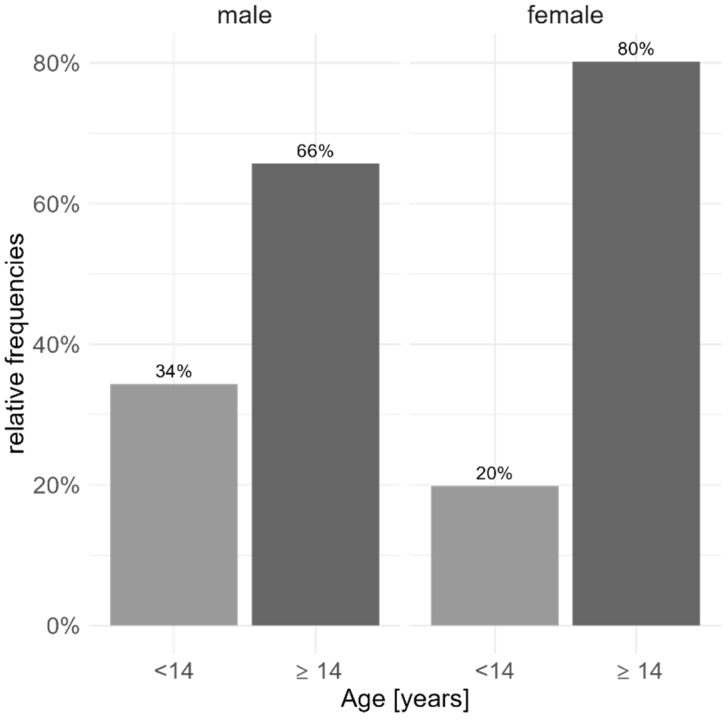
The sample included a higher proportion of adolescent girls compared to adolescent boys.

At baseline, female patients were slightly older (*p* = 0.02) compared to male patients. Moreover, sex was associated with the number of children/adolescents in the sample (*p* = 3.802 × 10^−5^, Cohen’s ω: 0.159); the sample included a higher proportion of adolescent girls compared to adolescent boys (Figure 1).

Female patients had less weight (*p* = 0.004) compared to male patients. BMI did not differ between the groups (*p* = 0.42). Also, the severity of illness did not differ between the groups (*p* = 0.702).

Diagnoses differed significantly between male and female patients (*p* < 0.001, Cohen’s ω: 0.569). Post hoc test showed that moderate depressive episodes (F32.1) and anorexia nervosa (F50.0) were significantly more often diagnosed in young females, while boys were diagnosed significantly more often with hyperkinetic disorders (F90.0/F90.1) and atypical autism (F84.1).

Concerning the last visit of the patients with available TDM, the number of medications did not differ significantly between boys and girls overall (*p* = 0.052). Nevertheless, to further investigate this effect, linear regression analysis, including the interaction age*sex as a confounder, revealed that age was associated with the number of medications (*p* = 0.022, ß = 0.187, CI = 0.027–0.346). Adolescents received more medications than children (adolescents: (mean, SD) 1.34, 0.58; children: 1.19, 0.43).

Regarding children and adolescents separately, the number of medications did not differ between adolescent boys and girls (*p* = 0.548). In contrast, among children under 14 years, girls received significantly more medications than boys (girls: 1.25 ± 0.47; boys: 1.11 ± 0.36; *p* = 0.03; r: 0,12; number of medication (mean ± sd)).

Treatment outcomes in the total sample were not associated with sex (*p* = 0.227). Also, in multinomial regression analysis, no covariate was associated with the probability for treatment response. ADEs were also not associated with sex (*p* = 0.556). Furthermore, in multinomial regression analysis analyses, no covariate was associated with the probability for ADEs.

### 3.2. Drug Specific Analyses

In these analyses, the most recent serum concentration determination per substance per patient was included irrespective of the study time point.

Due to possibly altered pharmacokinetic behavior in patients with anorexia nervosa, because of the altered hormonal status, as well as low body weight, these patients were excluded from drug specific analyses.

Patients’ characteristics, doses, serum concentrations, CD, MPR (if applicable), treatment outcome and ADEs for each drug are summarized in Supplement Table S1.

Serum concentration data were available for fluoxetine (204 patients: 48 male, 156 female), sertraline (90 patients: 33 male, 57 female), and mirtazapine (59 patients: 8 male, 51 female). For escitalopram and citalopram, serum concentration determinations in 49 patients (8 male, 41 female) and 32 patients (7 male, 25 female) were available.

Aripiprazole serum concentration data included 95 patients (47 male, 48 female), quetiapine 76 patients (18 male, 58 female), and olanzapine 40 patients (17 male, 23 female). For clozapine, serum concentration determinations in 6 patients (2 male, 4 female) were available; therefore, analyses were not possible due to limited sample size. Regarding risperidone, serum concentration determinations in 30 patients were available, but three were excluded from analysis for potential non-adherence (active moiety serum concentrations: 0 ng/mL). Thus, 27 patients on risperidone (21 male, 6 female) remained for analysis.

Detailed statistics were summarized in Supplement S1.

### 3.3. Pharmacokinetic Analyses

Active moiety serum concentrations of fluoxetine were significantly higher in female compared to male patients in the whole sample (*p* = 0.012). In age-specific analyses, in children, fluoxetine active moiety serum concentration did not differ (*p* = 0.400), but in adolescent patients, females showed higher serum concentrations compared to boys (*p* = 0.010). Serum concentrations of aripiprazole between male and female patients did not differ when summarizing children and adolescents; however, only focusing on children, aripiprazole serum concentrations were higher in girls compared to boys (*p* = 0.035), but in adolescent patients, serum concentrations did not differ between male and female patients (*p* = 0.071). For quetiapine, sertraline, olanzapine, mirtazapine, escitalopram, citalopram, and risperidone, we found no difference in serum concentrations and active moiety serum concentrations, respectively, for risperidone, between boys and girls in the whole sample, but also analyzing children/adolescents separately.

Looking into CD, linear regression analyses to correct for confounders (sex*age, weight, and CYP-inhibiting drugs (if relevant)), revealed that CD of aripiprazole, fluoxetine, N-desmethylfluoxetine and the active moiety of fluoxetine, quetiapine, sertraline, olanzapine, mirtazapine, escitalopram, citalopram, and risperidone were not affected by the sex of the patients in our sample. Nevertheless, weight was associated with CD of aripiprazole, fluoxetine, N-desmethylfluoxetine and the active moiety of fluoxetine, sertraline, and citalopram (for details see Supplement S1).

For fluoxetine and risperidone analyses on MPR were possible; however, no confounder was associated with MPR.

### 3.4. Pharmacodynamic Analyses

Looking into pharmacodynamics, multinomial logistic regression analyses to correct for confounders (for details see Supplement S1) showed that in the fluoxetine sample, in patients diagnosed with F3x, female sex (*p* = 0.04) was associated with the probability for very good therapy response, as well as with moderate treatment response (*p* = 0.02).

Treatment outcome, separated for F2x, F3x, and F4x diagnoses was not associated with sex in the aripiprazole, quetiapine, sertraline, olanzapine, mirtazapine, escitalopram, and citalopram samples. Additionally, ADEs were not associated with sex for aripiprazole, fluoxetine, quetiapine, sertraline, olanzapine, mirtazapine, escitalopram, and citalopram. For risperidone, no pharmacodynamic analyses were conducted due to low sample size in the groups (F2x, F3x, F4x).

## 4. Discussion

Our findings suggest that, in our sample, sex did not affect the pharmacokinetics of psychiatric drugs in children/adolescents when considering confounding parameters (age, weight, and CYP-affecting drugs). However, treatment outcome in drug-specific analyses of fluoxetine was associated with sex, with higher probability for a better outcome in female patients diagnosed with mood (affective) disorders.

The majority of participants included in the study were female (66.5%). On average, female youths were older than males (14.9 vs. 14.1 years) and generally had lower body weight, likely influenced by the high prevalence of anorexia nervosa among female patients. This sex distribution is expected, given the higher incidence of emotional and eating disorders in adolescent females compared to males, as noted in previous studies [31,32]. Additionally, the finding that younger boys are more frequently diagnosed with expansive disorders aligns with typical sex patterns in child and adolescent psychiatric disorders [31,33]. Consequently, the sample can be considered in this respect to be representative of the population treated at specialized child and adolescent psychiatric clinics in the three participating countries. Furthermore, the observation that adolescents received AD and AP treatment more frequently than children aligns with findings from other studies [34,35]. Within the adolescent subgroup, the number of medications did not differ between boys and girls, whereas in children, the number of medications in girls was higher compared to boys. One plausible explanation is that depressive disorders, which are more commonly diagnosed in girls even at a younger age, often require more complex psychopharmacological treatments—such as antidepressants, sometimes augmented with antipsychotics or other agents. In contrast, ADHD, which is more frequently diagnosed in boys, is usually managed with a single stimulant medication in monotherapy. Furthermore, antipsychotics—occasionally used to manage externalizing disorders—are prescribed more cautiously and less frequently in younger children, which may further reduce overall medication use in boys at this age.

In this study, overall treatment outcome and the occurrence of ADEs, irrespective of the drug, were not associated with sex. This contrasts with findings in adults, where female sex has been identified as a risk factor for ADEs. In adults, higher rates of ADEs under AD treatment have been reported for women [20,21]. On the other hand, selective serotonin reuptake inhibitors appear to be more effective in women than in men [1]. A recent study involving over 900 participants with depressive disorders, including children aged 12 years and older, found that girls and women benefited more from AD treatment compared to boys and men [36]. In our drug-specific analyses, when including confounders (age, weight, serum concentration of the patient, and number of medication), female sex was associated with more favorable treatment responses to fluoxetine in patients diagnosed with mood (affective) disorders, which aligns with previous results [36]. In line with our findings on no sex-dependent frequencies of ADEs, a study investigating prolactin elevation as an ADE of AP treatment in children and adolescents reported similar levels of prolactin elevation between sexes, along with cases showing no changes during antipsychotic treatment [37].

Serum concentrations of aripiprazole differed between boys and girls under 14 years, with higher concentrations observed in girls. However, this difference disappeared after adjusting for dose. Including confounding parameters showed that weight and the intake of CYP2D6-inhibiting comedications was associated with CD of aripiprazole. Therefore, higher CD levels were observed in patients treated with concomitant CYP2D6 inhibitors. Aripiprazole pharmacokinetics, thus, was not affected by sex in our study. This result is in line with results in adult patients, where no differences between male and female patients were reported [38]. In addition, one study in adolescent patients also reported no differences between male and female patients, aged 13.5 to 21.6 years [39]. However, the authors did not consider weight but the BMI of the patients [39], even if, for dosing in children/adolescents, weight is considered.

Consistent with other studies, we observed a sex difference in active moiety serum concentrations of fluoxetine, with adolescent females showing higher levels than adolescent males [11,12]. However, in our study, no such sex difference was found in children treated with fluoxetine. The observed difference in active moiety serum concentrations of fluoxetine between adolescent females and males, but not in younger children, could be due to several factors related to biological and developmental changes during adolescence.

First, hormonal differences become more pronounced during adolescence, especially with the increase in estrogen in females, which can affect liver enzyme activity responsible for drug metabolism, potentially leading to higher fluoxetine concentrations in females. Second, body composition changes, such as an increased body fat percentage in adolescent females compared to males, might influence the distribution and storage of lipophilic drugs like fluoxetine, resulting in higher serum concentrations. Third, differences in metabolic rates that emerge as children progress into adolescence could also alter pharmacokinetic variables. Adolescents often show variable rates of drug clearance due to differences in the maturation of the cytochrome P450 enzyme system, particularly CYP2D6, which is involved in fluoxetine metabolism. Lastly, behavioral factors, such as adherence to medications, diet, or comedication use, could also differ by sex and age group, potentially influencing drug levels. Thus, taken together, the absence of a sex difference in children might reflect a more homogeneous metabolic rate, hormonal status, and body composition in younger age groups before the onset of puberty. Further research could help clarify the roles that these physiological and developmental factors play in influencing fluoxetine metabolism across different age groups and between sexes.

Looking into CD, weight was associated with CD of fluoxetine, N-desmethylfluoxetine, and the active moiety but not sex (for details see Supplement S1 [40,41,42]). This is supported by previous results in adults, where no difference in CD of the active moiety was found between men and women [7].

Regarding risperidone, considering confounding factors, sex was not associated with CD of risperidone, 9OH-risperidone, and the active moiety. This result is supported by a previous study conducted by our research group, which found no sex differences in children and adolescents treated with risperidone [5].

Beside fluoxetine, CD of sertraline and citalopram were also associated with the weight of the patients (for details see Supplement S1 [40,41,42]); thus, in children/adolescents, possibly weight has to be considered for dose adjustment rather than the sex of the patients.

Interestingly, in contrast to findings in adult populations, we did not observe significant pharmacokinetic differences between sexes in our child/adolescent sample, when considering confounding parameters (age, weight, and CYP-affecting comedications). One possible explanation for this finding is that adolescence is a period of dynamic biological and hormonal changes, leading to high interindividual variability that may mask more demarcated pharmacokinetic patterns observed in adults. Moreover, pharmacokinetic differences are reported in adults due to higher doses being frequently used than in younger people who are often at an earlier stage in the illness. Differences in study design, sample size, or drug exposure levels may also contribute to these discrepancies.

These results highlight the need for further investigations into sex-specific pharmacological effects in child and adolescent psychiatry to optimize treatment strategies.

## 5. Strengths and Limitations

The strengths and limitations of the TDM Vigil study have been discussed in detail elsewhere [10]. The findings of this study should be interpreted with consideration of several limitations. As this was an exploratory study, we did not apply corrections for multiple comparisons (e.g., Bonferroni or Benjamini–Hochberg), which increases the risk of false-positive findings and should be taken into account when interpreting the results. Due to the small sample size in certain subgroups (e.g., risperidone in females), the findings should be interpreted as being exploratory.

Furthermore, information on pubertal status or hormonal levels was not available, which prevented additional analyses to explore age- and sex-group differences in the results.

Caution is warranted when interpreting the results related to treatment outcomes, as the sample size was small and heterogeneous, limiting the ability to assess sex differences for the individual AD and AP. Moreover, the CGI scale was used for illness severity and treatment response, even if disorder-specific outcome scales would have offered a more nuanced assessment. Nevertheless, the CGI scale provides a standardized measure that is applicable across diagnostic groups, allowing for consistent analysis in this heterogeneous clinical population. Additionally, the study was conducted in patients receiving care at specialized child and adolescent psychiatric centers, where they were closely and regularly monitored, assessed, and evaluated. Establishing causality is challenging in these complex psychiatric cases, where patients often receive multiple psychotropic medications, and treatment efficacy may also be influenced by concurrent treatments such a psychotherapy.

A key strength of this study is the use of ‘real-world data’ collected prospectively and in a standardized manner across 18 centers, with data quality ensured through controlled training and monitoring procedures. Unlike typical healthcare database samples, which are often less thoroughly characterized, our study offers a more comprehensive and precise dataset. Additionally, safety data in observational trials are usually inconsistently reported, but our approach addressed this gap. We conducted a multicenter trial using an internet-based patient registry, involving hospitals from three European countries, and one private specialist practice from Germany. This method of prospective, standardized monitoring of patients and medications has proven to be an effective approach for post-marketing surveillance in routine patient care. Furthermore, we included a heterogeneous patient population, reflective of clinical practice, without applying common exclusion criteria found in clinical trials, such as suicidality. Moreover, focusing on the drug-specific, pharmacokinetic analyses, patients diagnosed with anorexia nervosa were excluded in order to minimize potential bias due to altered pharmacokinetics in this specific population. In fact, a substantial number of patients (mainly females) in our sample were diagnosed with anorexia nervosa. To ensure the validity of the drug-specific analyses, we therefore decided to exclude these patients from that part of the analyses. While this decision may have slightly reduced statistical power, we believe this approach improved the internal validity and interpretability of the pharmacological findings.

## 6. Conclusions

In conclusion, our study investigated age and sex-related factors in the therapeutic outcomes of AD and AP in children and adolescents. Female sex was associated with a better treatment outcome in patients treated with fluoxetine.

In contrast, in our samples, pharmacokinetics did not differ between male and female patients in children and adolescents. Thus, we suggest that in children/adolescents, weight has to be considered for dose adjustment rather than the sex of the patients.

However, further research is essential to explore these influences and their implications for personalized treatment strategies.

## Figures and Tables

**Table 1:** Demographics and clinical characteristics of the patients.

	All Patients	Male	Female	*p* Value (Effect Size)
**Number of patients**	705	236	469	
**Age (mean; SD (min, max)) (Baseline)**	14.6, 2.2 (6, 18)	14.1, 2.9 (6, 18)	14.9, 1.7 (8, 18)	0.020 (r: 0.06)
**number < 14 years**	174	81	93	<0.001 (Cohen‘s ω: 0.159)
**number ≥ 14 years**	531	155	376	
**Weight [kg] (Baseline)**	55.7, 16.5 (19.9, 135)	58.6, 20.7 (19.9, 135)	54.3, 13.7 (21.4, 114.0)	0.004 (r: 0.07)
**BMI (Baseline)**	20.4, 4.6 (10.8, 40.3)	20.7, 4.8 (12.6, 40.3)	20.3, 4.5 (10.8, 37.4)	0.424
**Most common diagnoses according to the ICD 10 (N (relative number) (Baseline)**				<0.001 (Cohen’s ω: 0.569)
**Moderate depressive episode (F32.1)**	265 (0.229)	61 (0.158)	204 (0.265)	
**Anorexia nervosa (F50.0)**	111 (0.096)	1 (0.003)	110 (0.194)	
**Severe depressive episode without psychotic symptoms (F32.2)**	85 (0.073)	13 (0.040)	72 (0.158)	
**Social phobias (F40.1)**	49 (0.042)	25 (0.080)	24 (0.062)	
**Mixed obsessional thoughts and acts (F42.2)**	49 (0.042)	18 (0.063)	31 (0.086)	
**Disturbance of activity and attention (F90.0)**	41 (0.035)	26 (0.097)	15 (0.045)	
**Hyperkinetic conduct disorder (F90.1)**	33 (0.028)	31 (0.128)	2 (0.006)	
**Recurrent depressive disorder, current episode moderate (F33.1)**	32 (0.028)	6 (0.028)	26 (0.083)	
**Paranoid schizophrenia (F20.0)**	27 (0.023)	16 (0.078)	11 (0.038)	
**Post-traumatic stress disorder (F43.1)**	26 (0.022)	7 (0.037)	19 (0.069)	
**Bulimia nervosa (F50.2)**	22 (0.019)	0 (0.000)	22 (0.086)	
**Severe depressive episode with psychotic symptoms (F32.3)**	20 (0.017)	9 (0.049)	11 (0.047)	
**Emotionally unstable personality disorder (F60.3)**	20 (0.017)	2 (0.012)	18 (0.080)	
**Depressive conduct disorder (F92.0)**	19 (0.016)	7 (0.041)	12 (0.058)	
**Atypical anorexia nervosa (F50.1)**	14 (0.012)	1 (0.006)	13 (0.067)	
**Mild depressive episode (F32.0)**	11 (0.009)	5 (0.031)	6 (0.033)	
**Generalized anxiety disorder (F41.1)**	11 (0.009)	3 (0.019)	8 (0.046)	
**Predominantly compulsive acts [obsessional rituals] (F42.1)**	11 (0.009)	3 (0.019)	8 (0.048)	
**Atypical autism (F84.1)**	10 (0.009)	9 (0.059)	1 (0.006)	
**Severity of illness (CGI-S) (Baseline)**				0.702
**Extremely ill**	29	6	23	
**Severely ill**	234	84	150	
**Markedly ill**	331	107	222	
**Moderately ill**	78	27	52	
**Mildly ill**	6	2	4	
**Treatment outcome CGI-I (last visit)**				0.227
**no improvement**	21	5	16	
**low response**	43	13	30	
**moderate response**	72	20	52	
**very good response**	35	16	19	
**NA**	4	1	3	
**Adverse drug effects (last visit)**				0.556
**none**	89	30	59	
**mild**	71	21	50	
**moderate**	1	1	0	
**serious**	9	3	6	
**Number of substances (mean, SD (min, max)) (last visit)**	1.3, 0.6 (1, 4)	1.24, 0.48 (1, 3)	1.34, 0.58 (1, 4)	0.052
**TDM availability**				
**Antidepressants (first TDM per patient)**				
**Fluoxetine**	356	78	276	
**Sertraline**	177	64	109	
**Mirtazapine**	116	11	104	
**Escitalopram**	97	17	80	
**Citalopram**	74	10	64	
**Venlafaxine**	28	1	27	
**Fluvoxamine**	15	4	11	
**Doxepin**	7	0	7	
**Clomipramine**	5	5	0	
**Amitriptyline**	5	0	5	
**Trimipramine**	2	0	2	
**Antipsychotics**				
**Aripiprazole**	194	88	106	
**Quetiapine**	178	43	131	
**Olanzapine**	154	40	114	
**Risperidone**	46	37	8	
**Clozapine**	36	18	18	
**Pipamperone**	20	14	6	
**Amisulpride**	11	4	7	
**Haloperidol**	7	2	5	
**Melperone**	6	0	6	
**Tiapride**	3	3	0	
**Sulpiride**	2	2	0	
**Paliperidone**	**1**	**0**	**1**	
**Trazodone**	1	0	1	
**Anticonvulsants**				
**Valproic acid**	13	10	3	
**Levetiracetam**	5	0	2	
**Lamotrigine**	2	0	1	
**Topiramate**	2	2	0	
**ADHD-medication**				
**Atomoxetine**	1	0	1	

ICD10: International classification of diseases. ADHD: Attention-Deficit/Hyperactivity Disorder.

## Data Availability

The data presented in this study are available on request from the corresponding author. The data are not publicly available because these are sensitive health patient data.

## Supplementary Materials

The following supporting information can be downloaded at: https://www.mdpi.com/article/10.3390/pharmaceutics17080983/s1, Supplement S1: Table S1 Patients characteristics, doses, serum concentrations, dose-corrected serum concentrations, metabolite-to-parent ratio (MPR) (if applicable), treatment outcome and adverse drug effects in the respective drug sample. P values were reported for Mann-Whitney U test, and Fishers exact test comparing the items between males and females not considering confounders (no regression analyses). Weight was only compared in case of significant results in linear regression analysis. Significant values were bold. Table S2 Log P values describing lipophilicity of the drugs according to DrugBank [42] and the main CYP enzymes in the metabolism of the respective drugs according to Hiemke et al. [8]. Figure S1 Dose-corrected serum concentrations of N-desmethylfluoxetine and the active moiety of fluoxetine were higher in females compared to males. Figure S2 Dose-corrected serum concentrations of fluoxetine, N-desmethylfluoxetine and the active moiety of fluoxetine were higher in females compared to males in adolescent patients. Figure S3 Dose-corrected serum concentration of 9-hydroxyrisperidone was higher in girls compared to male patients. Figure S4 Dose-corrected serum concentration (CD) of 9-hydroxyrisperidone was higher in females compared to males in adolescent patients.
