# Supplementary material for: Sex-Specific Differences in Antidepressant and Antipsychotic Treatment Outcomes and Serum Levels in Children and Adolescents
Source: Pharmaceutics. 2025 Jul 30;17(8):983. doi: 10.3390/pharmaceutics17080983 (PMC12389296; doi:10.3390/pharmaceutics17080983)
Supplement: Supplementary file 1 [file pharmaceutics-17-00983-s001.zip › pharmaceutics-3741860-supplementary.pdf]

Supplement S1

To

Sex specific differences in the antidepressant- and antipsychotic treatment outcome and serum levels in children and adolescents

I. *Table S1 Patients characteristics, doses, serum concentrations, dose-corrected serum concentrations, metabolite-to-parent ratio (MPR) (if applicable), treatment outcome and adverse drug effects in the respective drug sample. P values were reported for Mann-Whitney U test, and Fishers exact test comparing the items between males and females not considering confounders (no regression analyses). Weight was only compared in case of significant results in linear regression analysis. Significant values were bold.*

					children			adolescents		
	**all patients**	**male**	**female**	**p value**	**male**	**female**	**p value**	**male**	**female**	**p value**
Fluoxetine	204	48	156		16	29		32	127	
**Age (mean, SD (min, max))**	14.7, 1.9 (7, 18)	14.2, 2.4 (7, 17)	14.9, 1.6 (9, 18)		11.3, 1.6 (7, 13)	12.3, 1.0 (9, 13)		15.6, 1.1 (14, 17)	15.4, 1.1 (14, 18)	
**Children/Adolescents**	45/159	16/32	29/127		16/0	29/0		0/32	0/127	
**Weight (mean, SD (min, max)) [kg]**	58.7, 16.7 (24.4, 124.7)	63.6, 23.2 (24.4, 120.2)	57.2, 13.7 (27.2, 124.7)		45.1, 14.2 (24.4, 71.6)	51.0, 12.8 (27.2, 73.6)	0.140	72.9, 21.3 (38.9, 120.2)	58.6, 13.6 (36.6, 124.7)	**<0.001**
**Dose (mean, SD (min, max)) [mg]**	20.7, 6.6 (10, 60)	20.7, 7.2 (10, 50)	20.7, 6.5 (10, 60)		16.3, 4.3 (10, 20)	18.8, 4.4 (10, 30)		23.0, 7.3 (10, 50)	21.2, 6.8 (10, 60)	
**Serum concentration (mean, SD (min, max)) [ng/ml]**	134.3, 82.5 (17, 541)	124.8, 94.4 (23, 541)	137.2, 78.6 (17, 419)		135.6, 81.9 (28, 382)	141.6, 75.8 (33, 294)		119.4, 11.9 (23, 541)	136.2, 79.5 (17, 419)	
**Metabolite serum concentration (mean, SD (min, max)) [ng/ml]**	151.1, 84.6 (30, 914)	125.7, 61.7 (30, 326)	157.8, 89.2 (41, 914)		130.1, 67.2 (47, 281)	144.2, 56.2 (72, 322)		123.4, 59.6 (30, 326)	162.0, 94.8 (41, 914)	
**Active moiety serum concentration (mean, SD (min, max)) [ng/ml]**	281.6, 140.2 (62, 1312)	245.3, 125.6 (62, 603)	292.9, 142.9 (84, 1312)	**0.012**	265.7, 127.6 (108, 603)	275.9, 109.2 (122, 536)	0.400	235.1, 125.3 (62, 541)	296.8, 149.8 (84, 1312)	**0.010**
**CD (mean, SD (min, max))**	6.5, 3.2 (1.2, 19.1)	6.1, 3.6 (1.2, 19.1)	6.6, 3.1 (1.5, 18.1)	0.193	8.1, 3.7 (2.8, 19.1)	7.6, 3.8 (2.3, 16.1)	0.514	5.0, 3.1 (1.2, 11.9)	6.4, 2.9 (1.5, 18.1)	**0.008**
**$CD_{metabolite}$ (mean, SD (min, max))**	7.4, 3.3 (1.5, 24.8)	6.6, 3.3 (1.5, 16.3)	7.7, 3.3 (2.1, 24.8)	**0.04**	7.9, 3.1 (3.1, 14.1)	7.8, 2.9 (2.7, 14.6)	0.920	5.9, 3.2 (1.5, 16.3)	7.7, 3.3 (2.1, 24.8)	**0.004**
**$CD_{active\ moiety}$ (mean, SD (min, max))**	13.7, 5.1 (2.1, 30.2)	12.4, 5.9 (2.1, 30.2)	14.1, 4.7 (5.0, 26.5)	**0.03**	16.0, 5.1 (10.7, 30.2)	14.9, 5.1 (6.1, 22.9)	0.514	10.5, 5.5 (2.1, 26.5)	13.9, 4.6 (5.0, 26.5)	**<0.001**
**MPR**	1.4, 1.2 (0.2, 14.6)	1.4, 0.8 (0.4, 4.0)	1.5, 1.3 (0.2, 14.6)	0.578	1.2, 0.7 (0.4, 3.0)	1.3, 0.6 (0.3, 2.8)	0.474	1.5, 0.9 (0.5, 4.0)	1.5, 1.4 (0.2, 14.6)	0.947
**Treatment outcome**										
**no improvement**	15	3	12		0	1		3	11	
**low response**	65	14	51		5	12		9	39	

moderate response	86	17	69		3	11		14	58	
very good response	26	11	15		6	3		5	12	
NA	3	1	2		0	1		1	1	
**Adverse drug effects**		.								
none	125	34	91		11	15		23	76	
mild	62	10	52		4	10		6	42	
moderate	1	1	0		0	0		1	0	
serious	3	0	3		0	2		0	1	
Sertraline	90	33	57		13	17		20	40	
**Age (mean, SD (min, max))**	14.2, 2.4 (7, 18)	13.9, 2.9 (7, 18)	14.4, 2.1 (8, 17)		10.9, 2.0 (7, 13)	11.8, 1.5 (8, 13)		15.8, 1.2 (14, 18)	15.5, 1.1 (14, 17)	
**Children/Adolescents**	30/60	13/20	17/40		13/0	17/0		0/20	0/40	
**Weight (mean, SD (min, max)) [kg]**	56.4, 17.3 (20.3, 112)	62.3, 23.4 (20.3, 112)	52.9, 11.4 (25.9, 84.5)		41.6, 14.9 (20.3, 77.8)	46.0, 11.0 (25.9, 59.8)	0.202	75.8, 17.2 (54, 112)	55.8, 10.3 (39.8, 84.5)	**<0.001**
**Dose (mean, SD (min, max)) [mg]**	106.4, 49.8 (25, 250)	107.6, 50.2 (50, 250)	105.7, 50.0 (25, 225)		101.9, 49.4 (50, 200)	116.2, 50.0 (50, 200)		111.25, 51.6 (50, 250)	101.3, 50.0 (25, 225)	
**Serum concentration (mean, SD (min, max)) [ng/ml]**	41.2, 29.7 (4, 125)	38.2, 27.3 (7, 109)	43.0, 31.0 (4, 125)	0.500	39.9, 36.7 (7, 109)	46, 29.2 (7, 111)	0.233	37.1, 20.1 (15, 91)	41.8, 32.1 (4, 125)	0.913
**CD (mean, SD (min, max))**	0.4, 0.2 (0.05, 1.3)	0.4, 0.2 (0.05, 0.9)	0.4, 0.3 (0.05, 1.3)	0.558	0.4, 0.3 (0.05, 0.9)	0.4, 0.1 (0.14, 0.7)	0.490	0.4, 0.2 (0.1, 0.6)	0.4, 0.3 (0.1, 1.3)	0.906
**Treatment outcome**										
no improvement	4	0	4		0	1		0	3	
low response	21	5	16		3	3		2	13	
moderate response	45	17	28		8	9		9	19	
very good response	15	9	6		2	4		7	2	
NA	0	0	0		0	0		0	0	
**Adverse drug effects**										
none	51	21	30		8	11		13	19	
mild	32	9	23		4	6		5	17	
moderate	0	0	0		0	0		0	0	
serious	4	2	2		1	0		1	2	
Mirtazapine	59	8	51		0	3		8	48	
**Age (mean, SD (min, max))**	15.8, 1.2 (13, 18)	16.4, 1.2 (14, 18)	15.7, 1.2 (13, 17)			13, 0 (13)		16.4, 1.2 (14, 18)	15.9, 1.0 (14, 17)	
**Children/Adolescents**	3/56	0/8	3/48			3/0		0/8	0/48	
**Weight (mean, SD (min, max)) [kg]**	55.3, 10.3 (33.9, 81.4)	64.2, 9.8 (53.1, 79.4)	53.9, 9.7) 33.9, 81.4)			50.8, 14.6 (33.9, 59.3)		64.2, 9.8 (53.1, 79.4)	54.1, 9.5 (39.0, 81.4)	
**Dose (mean, SD (min, max)) [mg]**	17.7, 10.6 (7.5, 45)	16.9, 8.7 (7.5, 30)	17.8, 10.8 (7.5, 45)			7.5, 0 (7.5)		16.9, 8.7 (7.5, 30)	18.4, 10.9 (7.5, 45)	
**Serum concentration (mean, SD (min, max)) [ng/ml]**	22.5, 15.6 (2, 81)	21.0, 9.3 (6, 33)	22.7, 16.4 (2, 81)	0.715		15.3, 4.9 (12, 21)		21.0, 9.3 (6, 33)	23.2, 16.8 (2, 81)	0.779
**CD (mean, SD (min, max))**	1.3, 0.6 (0.3, 2.8)	1.4, 0.7 (0.4, 2.5)	1.3, 0.6 (0.3, 2.8)	0.595		2.0, 0.7 (1.6, 2.8)		1.4, 0.7 (0.4, 2.5)	1.3, 0.5 (0.3, 2.5)	0.497
**Treatment outcome**										

no improvement	6	0	6			0		0	6	
low response	15	4	11			0		4	11	
moderate response	20	1	19			2		1	17	
very good response	11	3	8			1		3	7	
NA	0	0	0			0		0	0	
**Adverse drug effects**										
none	20	4	16			0		4	16	
mild	29	4	25			3		4	22	
moderate	3	0	3			0		0	3	
serious	2	0	2			0		0	2	
Escitalopram	49	8	41		0	3		8	38	
**Age (mean, SD (min, max))**	15.5, 1.3 (12, 18)	16.5, 0.8 (15, 17)	15.3, 1.3 (12, 18)			12.7, 0.6 (12, 13)		16.5, 0.8 (15, 17)	15.5, 1.1 (14, 18)	
**Children/Adolescents**	3/46	0/8	3/38			3/0		0/8	0/38	
**Weight (mean, SD (min, max)) [kg]**	61.9, 17.2 (40.5, 146)	84.6, 27.5 (59, 146)	57.4, 9.7 (40.5, 85)			60.3, 17.0 (47.7, 79.6)		84.6, 27.5 (59, 146)	57.2, 9.3 (40.5, 85)	
**Dose (mean, SD (min, max)) [mg]**	15.5, 8.6 (5, 60)	14.4, 5.0 (10, 20)	15.7, 9.1 (5, 60)			16.7, 10.4 (5, 25)		14.4, 5.0 (10, 20)	15.7, 9.2 (5, 60)	
**Serum concentration (mean, SD (min, max)) [ng/ml]**	22.8, 19.0 (6, 103)	19.9, 10.9 (8, 40)	23.4, 20.2 (6, 103)	0.989		15, 9.8 (7, 26)		19.9, 10.9 (8, 40)	24.0, 20.8 (6, 103)	0.942
**CD (mean, SD (min, max))**	1.6, 1.1 (0.3, 5.2)	1.4, 0.6 (0.7, 2)	1.6, 0.1 (0.3, 5.2)	0.946		1.0, 0.4 (0.6, 1.4)		1.4, 0.6 (0.7, 2)	1.7, 1.2 (0.3, 5.2)	0.805
**Treatment outcome**										
no improvement	2	0	2			0		0	2	
low response	14	2	12			0		2	12	
moderate response	19	1	18			1		1	17	
very good response	9	1	8			2		1	6	
NA	2	1	0			0		1	0	
**Adverse drug effects**										
none	27	3	24			3		3	21	
mild	14	2	12			0		2	12	
moderate	1	0	1			0		0	1	
serious	3	0	3			0		0	3	
Citalopram	32	7	25		3	6		4	19	
**Age (mean, SD (min, max))**	14.5, 2.0 (9, 17)	14, 3.4 (9, 17)	14.6, 1.5 (12, 17)		10.7, 2.1 (9, 13)	12.5, 0.6 (12, 13)		16.5, 1 (15, 17)	15.3, 1 (14, 17)	
**Children/Adolescents**	9/23	3/4	6/19		3/0	6/0		0/4	0/19	
**Weight (mean, SD (min, max)) [kg]**	57.3, 17.4 (28.5, 96.2)	57.2, 22.3 (28.5, 81.5)	57.3, 16.3 (35.4, 96.2)		44.8, 26.2 (28.5, 75)	55.9, 10.0 (47.3, 72.3)		66.6, 16.4 (44.2, 81.5)	57.7, 18.1 (35.4, 96.2)	
**Dose (mean, SD (min, max)) [mg]**	25.3, 11.6 (5, 40)	25.7, 11.3 (10, 40)	25.2, 11.9 (5, 40)		16.7, 5.8 (10, 20)	31.7, 9.8 (20, 40)		32.5, 9.6 (20, 40)	23.2, 11.9 (5, 40)	
**Serum concentration (mean, SD (min, max)) [ng/ml]**	58.7, 31.5 (10, 135)	50, 34.4 (17, 111)	61.1, 31.0 (10, 135)	0.316	50.3, 30.0 (32, 85)	72.5, 39.8 (29, 135)	0.697	49.8, 42.1 (17, 111)	57.5, 28.0 (10, 106)	0.516

**CD (mean, SD (min, max))**	2.6, 1.6 (0.6, 9.4)	2.1, 1.3 (0.6, 4.3)	2.7, 1.7 (1.0, 9.4)	0.386	3.1, 1.3 (1.7, 4.3)	2.3, 1.1 (1.5, 4.5)	0.517	1.5, 1.0 (0.6, 2.8)	2.8, 1.9 (1.0, 9.4)	0.074
**Treatment outcome**										
**no improvement**	2	0	2		0	0		0	2	
**low response**	10	3	7		2	1		1	6	
**moderate response**	10	2	8		1	2		1	6	
**very good response**	7	2	5		0	3		2	2	
**NA**	1	0	1		0	0		0	1	
**Adverse drug effects**										
**none**	16	6	10		2	6		4	4	
**mild**	13	1	12		1	0		0	12	
**moderate**	0	0	0		0	0		0	0	
**serious**	0	0	0		0	0		0	0	
Aripiprazole	95	47	48		17	12		30	36	
**Age (mean, SD (min, max))**	14.4, 2.5 (7, 18)	14.0, 3.0 (7, 17)	14.8, 1.9 (10, 18)		10.6, 2.2 (7, 13)	12.2, 1.1 (10, 13)		16.0, 1.0 (14, 17)	15.7, 1.1 (14, 18)	
**Children/Adolescents**	29/66	17/30	12/36		17/0	12/0		0/30	0/36	
**Weight (mean, SD (min, max)) [kg]**	61.9, 18.2 (24.7, 112.0)	62.6, 20.8 (24.7, 112.0)	61.2, 15.3 (26.9, 91.4)		49.2, 22.1 (24.7, 100)	52.5, 15.7 (26.9, 76.9)	0.39	70.2, 16.0 (32.6, 112.0)	64.1, 14.2 (43.6, 91.4)	0.089
**Dose (mean, SD (min, max)) [mg]**	9.8, 6.3 (2, 30)	12.6, 7.6 (3, 30)	9.6, 5.6 (2, 30)		6.2, 4.8 (2.5, 20)	8.2, 5.0 (2, 20)		12.2, 7.4 (2.5, 30)	9.9, 5.8 (2.5, 30)	
**Serum concentration (mean, SD (min, max)) [ng/ml]**	160.1, 115.8 (12, 571)	164.2, 124.3 (22, 571)	156.1, 108.1 (12, 451)	0.888	104.2, 77.9 (22, 291)	185.8, 120.8 (13, 451)	**0.035**	198.2, 133.5 (33, 571)	146.2, 103.5 (12, 449)	0.071
**CD (mean, SD (min, max))**	17.1, 8.3 (2.4, 57.1)	17.7, 8.9 (4.7, 57.1)	16.5, 7.6 (2.4, 38.1)	0.642	18.0, 7.6 (8.4, 32.4)	21.5, 7.9 (6.5, 35.1)	0.250	17.5, 9.7 (4.7, 57.1)	14.8, 6.9 (2.4, 38.1)	0.216
**Treatment outcome**										
**no improvement**	9	6	3		1	2		5	1	
**low response**	22	12	10		2	2		10	8	
**moderate response**	40	19	21		12	4		7	17	
**very good response**	20	8	12		1	3		7	9	
**NA**	1	0	1		0	1		0	0	
**Adverse drug effects**										
**none**	44	18	26		7	10		11	16	
**mild**	44	25	19		8	0		17	19	
**moderate**	1	1	0		0	0		1	0	
**serious**	1	1	0		1	0		0	0	
Quetiapine	76	18	58		1	12		17	46	
**Age (mean, SD (min, max))**	15.0, 1.6 (12, 17)	15.6, 1.2 (13, 17)	14.9, 1.6 (12, 17)		13	12.4, 0.5 (12, 13)		15.8, 1.1 (14, 17)	15.5, 1.2 (14, 17)	
**Children/Adolescents**	13/63	1/17	12/46		1/0	12/0		0/17	0/46	
**Weight (mean, SD (min, max)) [kg]**	62.6, 13.2 (37.3, 99.4)	69.3, 13.5 (42.4, 99.4)	60.5, 12.4 (37.3, 94.7)		76.3	61.2, 13.6 (37.3, 83.3)		68.9, 13.8 (42.4, 99.4)	60.3, 12.3 (43.4, 94.7)	
**Dose (mean, SD (min, max)) [mg]**	263.5, 198.4 (25, 800)	361.1, 217.3 (50, 650)	233.2, 183.8 (25, 800)		550	270.8, 143.8 (50, 600)		350, 218.7 (50, 650)	223.4, 193.0 (25, 800)	

**Serum concentration (mean, SD (min, max)) [ng/ml]**	117.3, 139.9 (3, 678)	130.8, 119.2 (4, 432)	113.0, 146.4 (3, 678)	0.219	234	93.4, 101.4 (25, 374)	0.229	124.8, 120.0 (4, 432)	118.2, 156.6 (3, 678)	0.329
**CD (mean, SD (min, max))**	0.6, 1.2 (0.03, 10.5)	0.4, 0.3 (0.1, 1.0)	0.7, 1.4 (0.03, 10.5)	0.337	0.4	0.4, 0.5 (0.1, 1.9)	0.688	0.4, 0.3 (0.08, 1.0)	0.7, 1.5 (0.03, 10.5)	0.168
**Treatment outcome**										
**no improvement**	9	1	8			3		1	5	
**low response**	22	4	18			3		4	15	
**moderate response**	18	5	13			3		5	10	
**very good response**	13	3	10			1		3	9	
**NA**	0	0	0			0		0	0	
**Adverse drug effects**										
**none**	27	4	23			8		4	15	
**mild**	34	9	25			2		9	23	
**moderate**	0	0	0			0		0	0	
**serious**	2	1	1			1		1	0	
Olanzapine	40	17	23		1	5		16	18	
**Age (mean, SD (min, max))**	15.2, 1.8 (10, 17)	15.6, 1.8 (10, 17)	14.8, 1.8 (10, 17)		10	12.0, 0.7 (11, 13)		15.9, 1.1 (14, 17)	15.6, 1.1 (14, 17)	
**Children/Adolescents**	6/34	1/16	5/18		1/0	5/0		0/16	0/18	
**Weight (mean, SD (min, max)) [kg]**	57.5, 15.9 (28.3, 86.5)	69.4, 12.2 (45.9, 86.5)	48.7, 12.2 (28.3, 79.5)		45.9	39.9, 8.0 (28.3, 46.2)		70.9, 10.9 (52.2, 86.5)	51.8, 11.6 (37.9, 79.5)	
**Dose (mean, SD (min, max)) [mg]**	10.7, 6.7 (2.5, 30)	13.6, 7.0 (5, 30)	8.5, 5.5 (2.5, 20)		10	6.5, 3.4 (2.5, 10)		13.8, 7.2 (5, 30)	9.0, 6.0 (2.5, 20)	
**Serum concentration (mean, SD (min, max)) [ng/ml]**	32.7, 24.5 (5, 134)	39.5, 30.2 (6, 134)	27.7, 18.6 (5, 85)	0.155	30	26.4, 15.1 (7, 45)	1.0	40.1, 31.1 (6, 134)	28.1, 19.8 (5, 85)	0.157
**CD (mean, SD (min, max))**	3.3, 1.6 (0.6, 8.9)	3.2, 2.0 (0.6, 8.9)	3.4, 1.3 (1.2, 6.8)	0.359	3	3.9, 0.7 (2.8, 4.5)	0.553	3.2, 2.0 (0.6, 8.9)	3.3, 1.4 (1.2, 6.8)	0.605
**Treatment outcome**										
**no improvement**	6	2	4		0	1		2	3	
**low response**	13	4	9		1	4		4	5	
**moderate response**	12	6	6		0	0		5	6	
**very good response**	6	3	3		0	0		3	3	
**NA**	1	0	1		0	0		0	1	
**Adverse drug effects**										
**none**	12	4	8		1	1		3	7	
**mild**	21	9	12		0	4		9	8	
**moderate**	0	0	0		0	0		0	0	
**serious**	4	2	2		0	0		2	2	
Risperidone	27	21	6		8	1		13	5	
**Age (mean, SD (min, max))**	13.7, 3.1 (7, 17)	13.2, 3.3 (7, 17)	15.3, 1.8 (12, 17)		9.5, 2.1 (7, 13)	12		15.5, 0.9 (14, 17)	16, 0.7 (15, 17)	
**Children/Adolescents**	9/18	8/13	1/5		8/0	1/0		0/13	0/5	
**Weight (mean, SD (min, max)) [kg]**	58.5, 16.9 (23.1, 84.5)	58.5, 17.9 (23.1, 77)	58.3, 14.4 (43, 84.5)		40.6, 16.2 (23.1, 74.4)	47.8		69.6, 5.6 (60.3, 77)	60.4, 15.0 (43, 84.5)	

**Dose (mean, SD (min, max)) [mg]**	2.0, 1.3 (0.5, 6)	1.9, 1.3 (0.5, 6)	2.25, 1.4 (1, 4)		1.1, 0.6 (0.5, 2.5)	3		2.4, 1.4 (1, 6)	2.1, 1.5 (1, 4)	
**Serum concentration (mean, SD (min, max)) [ng/ml]**	9.4, 13.8 (0, 46)	10.2, 15.4 (0, 46)	6.5, 5.0 (0, 12.8)		2.25, 1.5 (0, 4)	8		15.2, 18.1 (0, 46)	6.2, 5.5 (0, 12.8)	
**Metabolite serum concentration (mean, SD (min, max)) [ng/ml]**	9.5, 7.0 (0, 24)	9, 6.7 (0, 22)	11.25, 8.8 (5, 24)		4, 1.4 (3,5)	24		9.9, 6.9 (0, 22)	7, 2.7 (5, 10)	
**Active moiety serum concentration (mean, SD (min, max)) [ng/ml]**	15.4, 16.3 (2, 55)	20.1, 19.7 (3, 55)	17, 12 (5, 32)	0.637	3.25, 0.9 (2, 5)	32	0.138	23, 20.2 (3, 55)	12, 8.2 (5, 21)	0.217
**CD (mean, SD (min, max))**	4.9, 6.4 (0, 23)	5.3, 7.0 (0, 23)	3.6, 3.9 (0, 11)	0.836	1.7, 1.7 (0, 4)	2.7	0.843	7.1, 8.4 (0, 23)	3.8, 4.3 (0, 11)	0.583
**$CD_{metabolite}$ (mean, SD (min, max))**	4.6, 2.3 (0, 10)	3.8, 1.6 (0, 6.3)	7.3, 2.2 (5, 10)	**0.012**	4, 1.4 (3, 5)	8	0.667	3.8, 1.7 (0, 6.3)	7, 2.7 (5, 10)	**0.040**
**$CD_{active\ moiety}$ (mean, SD (min, max))**	7.8, 7.2 (0.9, 27.5)	7.6, 7.3 (1.6, 27.5)	8.5, 7.2 (0.9, 21)	0.726	3.3, 1.1 (1.6, 5)	10.7	0.166	10.3, 8.3 (3, 27.5)	8.0, 8.0 (0.9, 21)	0.490
**MPR**	1.2, 1.1 (0.2, 3)	0.95, 1.1 (0.2, 3)	1.8, 1.1 (0.9, 3)	0.193	NA	3		0.95, 1.1 (0.2, 3)	1.2, 0.4 (0.9, 1.5)	0.402
**Treatment outcome**										
**no improvement**	4	3	1		0	1		3	0	
**low response**	9	8	1		4	0		4	1	
**moderate response**	7	6	1		3	0		3	1	
**very good response**	3	2	1		0	0		2	0	
**NA**	0	0	0		0	0		0	0	
**Adverse drug effects**										
**none**	5	4	1		3	0		1	1	
**mild**	16	13	3		4	1		9	2	
**moderate**	2	2	0		0	0		2	0	
**serious**	0	0	0		0	0		0	0	

II. Detailed statistical analyses - Results

Aripiprazole

In summary, concentration determinations in 95 patients (47 male, 48 female) were available.

Age independently, serum concentration between male and female patients did not differ (Mann-Whitney U test p=0.888); looking into children and adolescents, in children, serum concentration differed (p=0.035) with higher serum concentrations in female patients, but in adolescent patients serum concentrations did not differ between male and female patients (p=0.071).

Irrespective of the age, CD of aripiprazole in male and female patients did not differ (Mann-Whitney U test, p=0.642). Also, investigating children and adolescent patients separately, CD did not differ between male and female patients (children: p= 0.250; adolescents=0.216). In linear regression analysis, CD was associated with weight of the patients (p=0.018; ß=-0.117; CI=-0.22 - -0.02) and intake of CYP2D6 inhibiting drugs (p=0.027; ß=7.4; CI=0.8 – 13.9). The interaction sex*age just missed significance (female sex* adolescent age (>=14 years); p=0.056; ß=-6.84; CI=-13.84 – 0.17)

Weight was not different between adolescent males and females (p=0.089), and not in children (p=0.39). In correlation test (spearman correlation) weight was associated with CD (p=0.01, rho=-0.265). Therefore, CD decreased with increasing weight.

Thirty-four patients were diagnosed with F2x. In these patients, treatment outcome was associated with sex (Fishers exact test, p=0.032); however, in post-hoc group comparisons (pairwise Fisher test) were not significant. In multinomial regression analysis, no confounder was associated with probability for response. Adverse drug effects (ADE) were not affected by sex (Fishers exact, p=0.842). Extending this analysis to multinomial logistic regression analysis, showed that none of the confounders (age*sex, serum concentration, weight, number of medication) was associated with ADE.

Twenty-seven patients were diagnosed with F3x. In these patients, treatment outcome was not associated with sex (Fishers exact test, p=1.0). In multinomial regression analysis, no confounder was associated with probability for response. Adverse drug effects (ADE) were not affected by sex (Fishers exact, p=1.0). Extending this analysis to multinomial logistic regression analysis, showed that none of the confounders (age*sex, serum concentration, weight, number of medication) was associated with ADE.

Seventeen patients were diagnosed with F4x. In these patients, treatment outcome was not associated with sex (Fishers exact test, p=0.906). In multinomial regression analysis, no confounder was associated with probability for response. Adverse drug effects (ADE) were not

affected by sex (Fishers exact, p=0.302). Extending this analysis to multinomial logistic regression analysis, showed that none of the confounders (age*sex, serum concentration, weight, number of medication) was associated with ADE.

Fluoxetine

Serum concentration determinations in 204 patients (48 male, 156 female) were available.

Age independently, active moiety serum concentration between male and female patients differed significantly (Mann-Whitney U test, p=0.012) with higher concentrations in female patients. Looking into children and adolescents, in children, serum concentration did not differ (p=0.400), but in adolescent patients females were treated with higher serum concentrations compared to male patients (p=0.010).

Irrespective of the age, CD of fluoxetine in male and female patients did not differ (Mann-Whitney U test, p=0.193). CD of the active metabolite N-desmethylfluoxetine and the active moiety was higher in females compared to males (p=0.04; p=0.03) (Figure S1).

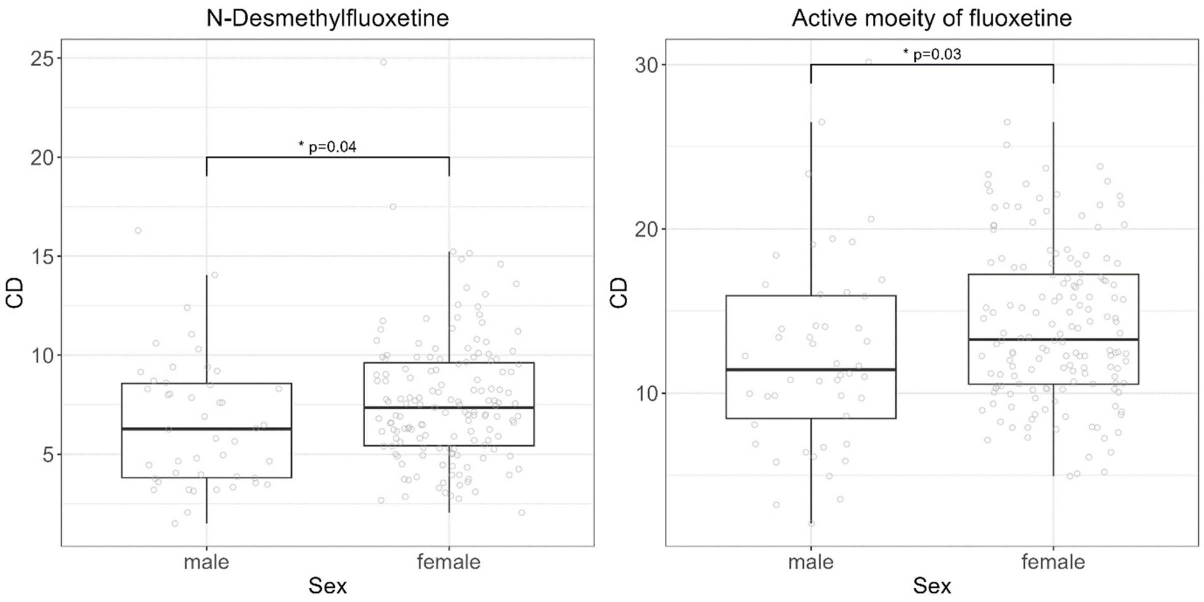

Figure S1 Dose-corrected serum concentrations of N-desmethylfluoxetine and the active moiety of fluoxetine were higher in females compared to males.

In children, CD of fluoxetine, N-desmethylfluoxetine, and the active moiety did not differ between males and females (p=0.514, p=0.920, p=0.514). However, in adolescent patients, CD of fluoxetine, N-desmethylfluoxetine, and the active moiety differed between males and females, with higher CD in girls (p=0.008, p=0.004, p=0.0003) (Figure S2).

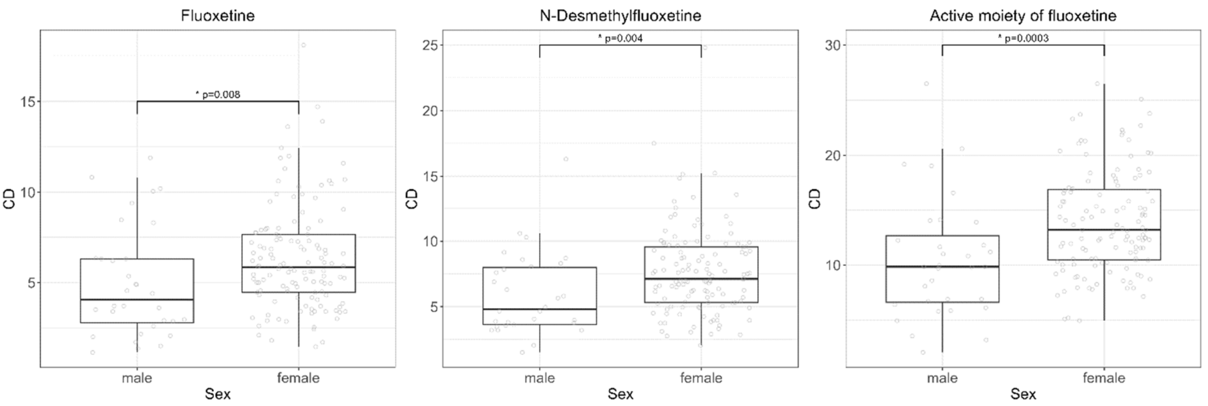

Figure S2 Dose-corrected serum concentrations of fluoxetine, N-desmethylfluoxetine and the active moiety of fluoxetine were higher in females compared to males in adolescent patients.

In linear regression analysis, CD of fluoxetine, N-desmethylfluoxetine and the active moiety was not associated with sex, or age, but with weight of the patients ($p=9.46*10^{-8}$; ß=-0.077; CI -105 - -0.050; $p=5.2*10^{-5}$, ß=-0.062, CI=-0.090 - -0.032; $p=1.15*10^{-9}$, ß=-0.136, CI=-0.178 - -0.094).

Weight also was different between males and females in adolescents (Mann-Whitney U test, $p=1.72*10^{-4}$), but not in children (p=0.14). In adolescent patients, male had higher weight compared to female patients. In correlation test (spearman correlation) weight was associated with CD of fluoxetine ($p=19.21*10^{-12}$, rho=-0.458), of N-desmethylfluoxetine ($p=1.35*10^{-5}$, rho=-0.307), and the active moiety ($p=9.612*10^{-13}$, rho=-0.478). Therefore, CD decreased with increasing weight.

MPR was not different between males and females in the whole fluoxetine sample (p=0.578), and not in children and adolescents (p=0.474, p=0.947). Also, in linear regression analysis no confounder was associated with MPR.

No patient diagnosed with F2x was treated with fluoxetine.

177 patients were diagnosed with F3x. In these patients, treatment outcome was not associated with sex (Fishers exact test, p=0.066). In multinomial regression analysis adolescent age (>=14 years) (p=0.02), and female sex was associated with the probability for very good therapy response (p=0.03), and female sex also was associated with moderate treatment response (p=0.01), but not age, the interaction sex*age, weight of the patients, or the number of medications. ADE were not affected by sex (Fishers exact, p=0.336). Extending this analysis to multinomial logistic regression analysis, showed that none of the confounders (age*sex, serum concentration, weight, number of medications) was associated with ADE.

Forty-eight patients were diagnosed with F4x. In these patients, treatment outcome was not associated with sex (Fishers exact test, p=0.663). In multinomial regression analysis, no confounder was associated with probability for response. ADE were not affected by sex (X^2-test, p=0.137). Extending this analysis to multinomial logistic regression analysis, showed that none of the confounders (age*sex, serum concentration, weight, number of medications) was associated with ADE.

Quetiapine

Serum concentration determinations in 76 patients (18 male, 58 female) were available. One patient was excluded due to non-adherence (serum concentration: 0 ng/ml).

Age independently, serum concentration between male and female patients did not differ (Mann-Whitney U test p=0.219). Looking into children and adolescents, in children and adolescents, serum concentration did not differ (p=0.229, p=0.329).

Irrespective of the age, CD of quetiapine in male and female patients did not differ (Mann-Whitney U test, p=0.337). In children and adolescents, CD of quetiapine did not differ between males and females (p=0.688, p=0.168). In linear regression analysis, CD of quetiapine was not associated with sex, age, or weight of the patients.

Twelve patients diagnosed with F2x were treated with quetiapine. In these patients, treatment outcome was not associated with sex (Fishers exact test, p=0.786). In multinomial regression analysis, no confounder was associated with probability for response. ADE were not affected by sex (Fishers exact, p=0.464). Extending this analysis to multinomial logistic regression analysis, showed that none of the confounders (age*sex, serum concentration, weight, number of medications) was associated with ADE.

Fifty patients diagnosed with F3x were treated with quetiapine. In these patients, treatment outcome was not associated with sex (Fishers exact test, p=0.087). In multinomial regression analysis, the number of medications was positively associated with the probability for very good treatment response (p=0.04; ß=1.99; CI=1.3 – 3.8). ADE were not affected by sex (Fishers exact, p=0.401). Extending this analysis to multinomial logistic regression analysis, showed that none of the confounders (age*sex, serum concentration, weight, number of medications) was associated with ADE.

Fourteen patients diagnosed with F4x were treated with quetiapine. In these patients, treatment outcome was not associated with sex (Fishers exact test, p=0.258). In multinomial regression analysis, no confounder was associated with probability for response. ADE were not affected by sex (Fishers exact, p=1.0). Extending this analysis to multinomial logistic

regression analysis, showed that none of the confounders (age*sex, serum concentration, weight, number of medications) was associated with ADE.

Sertraline

Serum concentration determinations in 90 patients (33 male, 57 female) were available.

Age independent serum concentration between male and female patients did not differ (Mann-Whitney U test p=0.500). Looking into children and adolescents, serum concentrations did not differ between males and females (p=0.233, p=0.913).

Irrespective of the age, CD of sertraline in male and female patients did not differ (Mann-Whitney U test, p=0.558). In children and adolescents, CD of sertraline did not differ between males and females (p=0.490, p=0.906). In linear regression analysis, CD of sertraline was not associated with sex, age, or CYP2C19 inhibiting drugs, but with weight of the patients (p=0.014, ß=-0.005, CI=-0.009 - -0.001).

Weight also was different between male and female patients in adolescents (Mann-Whitney U test, $p=8.512*10^{-6}$), but not in children (p=0.202). In adolescent patients, males had higher weight compared to female patients. In correlation test (spearman correlation) weight was associated with CD of sertraline (p=0.04, rho=-0.2123). Therefore, CD decreased with increasing weight.

Three patients diagnosed with F2x were treated with sertraline; therefore, further analyses were not conducted.

Fifty-three patients diagnosed with F3x were treated with sertraline. In these patients, treatment outcome was not associated with sex (Fishers exact test, p=0.723). In multinomial regression analysis, no confounder was associated with probability for response. ADE were not affected by sex (Fishers exact, p=0.074). Extending this analysis to multinomial logistic regression analysis, showed that none of the confounders (age*sex, serum concentration, weight, number of medications) was associated with ADE.

Forty-six patients diagnosed with F4x were treated with sertraline. In these patients, treatment outcome was not associated with sex (Fishers exact test, p=0.152). In multinomial regression analysis, no confounder was associated with probability for response. ADE were not affected by sex (Fishers exact, p=0.182). Extending this analysis to multinomial logistic regression analysis, showed that none of the confounders (age*sex, serum concentration, weight, number of medications) was associated with ADE.

Olanzapine

Serum concentration determinations in 40 patients (17 male, 23 female) were available.

Age independently, serum concentration between male and female patients did not differ (Mann-Whitney U test, p=0.155). Looking into children and adolescents, also, serum concentration did not differ (p=1.0, p=0.157).

Irrespective of the age, CD of olanzapine in male and female patients did not differ (Mann-Whitney U test, p=0.359). In children and adolescents, CD of olanzapine did not differ between males and females (p=0.553, p=0.605). In linear regression analysis, CD of olanzapine was not associated with sex, age, or weight of the patients. No patient was simultaneously treated with CYP1A2 affecting comedications.

Sixteen patients diagnosed with F2x were treated with olanzapine. In these patients, treatment outcome was not associated with sex (Fishers exact test, p=0.400). In multinomial regression analysis, no confounder was associated with probability for response (sex, serum concentration, weight, number of medications; all patients were older than 14 years). ADE were not affected by sex (Fishers exact, p=1.0). Extending this analysis to multinomial logistic regression analysis, showed that none of the confounders (age, serum concentration, weight, number of medications) was associated with ADE.

Also, 16 patients diagnosed with F3x were treated with olanzapine. In these patients, treatment outcome was not associated with sex (Fishers exact test, p=0.712). In multinomial regression analysis, no confounder was associated with probability for response. ADE were not affected by sex (Fishers exact, p=0.115). Extending this analysis to multinomial logistic regression analysis, showed that none of the confounders was associated with ADE.

Seven patients diagnosed with F4x were treated with olanzapine; therefore, further analyses were not conducted.

Mirtazapine

Serum concentration determinations in 59 patients (8 male, 51 female) were available.

Age independently, serum concentrations between male and female patients did not differ (Mann-Whitney U test, p=0.715). Looking into children and adolescents, in adolescents, serum concentration did not differ (p=0.779). Analysis on children were not available as no male younger than 14 years was treated with mirtazapine.

Irrespective of the age, CD of mirtazapine in male and female patients did not differ (Mann-Whitney U test, p=0.595). In adolescents, CD of mirtazapine did not differ between males and females (p=0.497). In linear regression analysis, CD of mirtazapine was associated with age (p=0.03, ß=-0.768, CI=-1.44 - -0.10). In children, therefore, CD was higher compared to adolescent patients. However, sex and weight were not associated with CD.

No patient diagnosed with F2x was treated with mirtazapine.

Fifty-four patients diagnosed with F3x were treated with mirtazapine. In these patients, treatment outcome was not associated with sex (Fishers exact test, p=0.111). In multinomial regression analysis, no confounder was associated with probability for response. ADE were not affected by sex (Fishers exact, p=1.0). Extending this analysis to multinomial logistic regression analysis, showed that none of the confounders was associated with ADE.

Twelve patients diagnosed with F4x were treated with mirtazapine. In these patients, treatment outcome was not associated with sex (Fishers exact test, p=0.127). In multinomial regression analysis, no confounder was associated with probability for response (sex, serum concentration, weight, number of medications; all patients were older than 14 years). ADE were not affected by sex (Fishers exact, p=470). Extending this analysis to multinomial logistic regression analysis, showed that none of the confounders was associated with ADE (sex, serum concentration, weight, number of medications; all patients were older than 14 years).

Escitalopram

Serum concentration determinations in 49 patients (8 male, 41 female) were available.

Age independently, serum concentration between male and female patients did not differ (Mann-Whitney U test, p=0.989). Looking into adolescents, serum concentration did not differ between males and females (p=0.942). Analysis on children were not available as no male younger than 14 years was treated with escitalopram.

Irrespective of the age, CD of escitalopram in male and female patients did not differ (Mann-Whitney U test, p=0.946). In adolescents, CD of escitalopram did not differ between males and females (p=0.805). In linear regression analysis, CD of escitalopram was not associated with sex, age, or weight of the patients.

Only three patients diagnosed with F2x was treated with escitalopram; thus, further analyses were not conducted.

Thirty-eight patients diagnosed with F3x were treated with escitalopram. In these patients, treatment outcome was not associated with sex (Fishers exact test, p=1.0). In multinomial regression analysis, no confounder was associated with probability for response. ADE were not affected by sex (Fishers exact, p=1.0). Extending this analysis to multinomial logistic regression analysis, showed that none of the confounders was associated with ADE.

Twelve patients diagnosed with F4x were treated with escitalopram. In these patients, treatment outcome was not associated with sex (Fishers exact test, p=0.727). In multinomial regression analysis, no confounder was associated with probability for response (sex, serum

concentration, weight, number of medications; all patients were older than 14 years). ADE were not affected by sex (Fishers exact, p=1.0). Extending this analysis to multinomial logistic regression analysis, showed that none of the confounders was associated with ADE (sex, serum concentration, weight, number of medications; all patients were older than 14 years).

Citalopram

Serum concentration determinations in 32 patients (7 male, 25 female) were available.

Age independently, serum concentration between male and female patients did not differ (Mann-Whitney U test, p=0.316). Looking into children and adolescents, serum concentration did not differ between males and females (p=0.697, p=0.516).

Irrespective of the age, CD of citalopram in male and female patients did not differ (Mann-Whitney U test, p=0.386). In children and adolescents, CD of citalopram did not differ between males and females (p=0.517, p=0.074). In linear regression analysis, CD of citalopram was not associated with sex, age, but with weight of the patients (p=0.042, ß=-0.035, CI=-0.069 - -0.001). No patient was simultaneously treated with a CYP2C19 inhibiting drug.

Weight did not differ between male and female patients in adolescents (p=0.351), neither in children (p=0.517). In correlation test (spearman correlation) weight was associated with CD of citalopram (p=0.003, rho=-0.512). Therefore, CD decreased with increasing weight.

Only one patient diagnosed with F2x was treated with citalopram.

Twenty-seven patients diagnosed with F3x were treated with citalopram. In these patients, treatment outcome was not associated with sex (Fishers exact test, p=0.871). In multinomial regression analysis, no confounder was associated with probability for response. ADE were not affected by sex (Fishers exact, p=0.098). Extending this analysis to multinomial logistic regression analysis, showed that none of the confounders was associated with ADE.

Nine patients diagnosed with F4x were treated with citalopram; thus, further analyses were not conducted.

Clozapine

Serum concentration determinations in 6 patients (2 male, 4 female) were available. Analyses were not possible due to limited patients.

Risperidone

Serum concentration determinations in 30 patients were available. Patients with non-adherence (N=3) were excluded from analysis (active moiety serum concentrations: 0 ng/ml). In consequence, 27 patients (21 male, 6 female) were available for analyses.

Age independently, active moiety serum concentration between male and female patients did not differ (Mann-Whitney U test, p=0.637). Looking into children and adolescents, active moiety serum concentration also did not differ (p=0.138, p=0.217) between male and female patients.

Irrespective of the age, CD of risperidone and the active moiety of risperidone in male and female patients did not differ (Mann-Whitney U test, p=0.836, p=0.726). In contrast, CD of 9OH-risperidone was higher in female compared to male patients (p=0.012) (Figure S3).

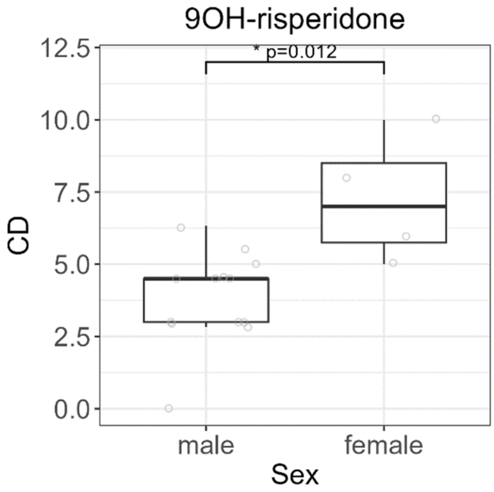

Figure S3 Dose-corrected serum concentration of 9-hydroxyrisperidone was higher in girls compared to male patients.

In children, CD of risperidone, 9OH-risperidone and the active moiety did not differ between males and females (p=0.843, p=0.667, p=0.166). In adolescent patients, CD of risperidone and the active moiety did not differ between males and females (p=0.583, p=0.490); in contrast CD of 9OH-risperidone was higher in female patients (p=0.040) (Figure S4).

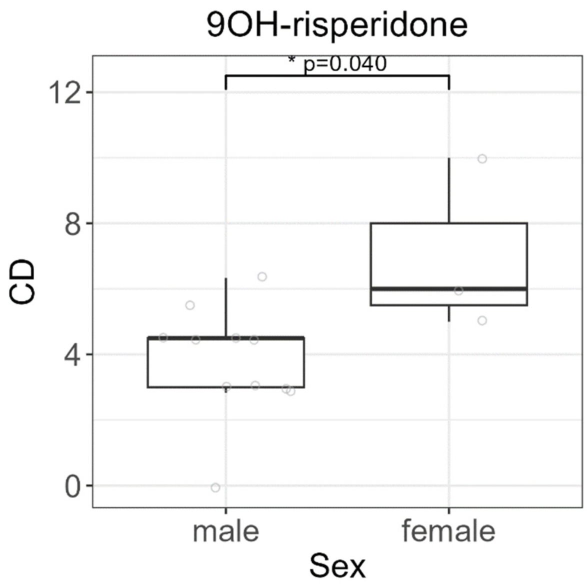

Figure S4 Dose-corrected serum concentration (CD) of 9-hydroxyrisperidone was higher in females compared to males in adolescent patients.

In linear regression analysis, CD of risperidone was not associated with sex, age, weight of the patients, nor with CYP2D6 inhibiting comedication. CD of 9OH-risperidone was associated with CYP2D6 inhibiting comedication (p=0.01; ß=-4.8; CI=-8.4 - -1.3). Therefore, CD of 9OH-risperidone was lower in patients treated with concomitant CYP2D6 inhibitors. CD of the active moiety was associated with age of the patients (p=0.05; ß=10.8; CI=0.1 – 21.6) with lower serum concentrations in adolescent patients compared to children.

MPR was not different between males and females in the whole sample (p=0.193), and not in adolescents (p=0.402). Also, in linear regression analysis no confounder was associated with MPR.

Eight patients diagnosed with F2x were treated with risperidone; thus, further analyses were not conducted.

Nine patients diagnosed with F3x were treated with risperidone; thus, further analyses were not conducted.

Three patients diagnosed with F3x were treated with risperidone; thus, further analyses were not conducted.

III. Discussion on weight

Body weight was associated with CD of aripiprazole, fluoxetine, N-desmethylfluoxetine and the active moiety of fluoxetine, sertraline, and citalopram with decreasing CD with increasing weight.

For these drugs that are lipophilic (positive log p values; Table S2) and distribute widely into body tissues, a higher body weight means a larger volume of distribution. This might lead to a dilution effect, causing lower CD. Moreover, as CD is inversely correlated to total clearance [8], the clearance of aripiprazole, fluoxetine, N-desmethylfluoxetine, sertraline, and citalopram may have increased with weight. It is known that clearance of drugs metabolized by CYP2D6, CYP2C9, CYP2C19 and CYP1A2 increased in obese patients [40]; however, also other target drugs are metabolized by these enzymes. Thus, we cannot explain why decreasing CD with increasing weight only is valid for aripiprazole, fluoxetine, N-desmethylfluoxetine and the active moiety of fluoxetine, sertraline, and citalopram, but not for quetiapine, olanzapine, mirtazapine, escitalopram, risperidone, or 9OH-risperidone, as these drugs also are lipophilic and metabolized by the specific enzymes (Table S2). However, in line with our results, in adult patients, BMI also was not associated with CD of escitalopram, mirtazapine, quetiapine and risperidone [41].

Table S2 Log P values describing lipophilicity of the drugs according to DrugBank [42] and the main CYP enzymes in the metabolism of the respective drugs according to Hiemke et al. [8]

	**N**	**Log P (lipophilicity)[4]**	**Metabolism (main CYP enzymes)[1]**
Aripiprazole	98	5.21	CYP2D6, CYP3A4
Fluoxetine	207	4.09	CYP2C9, CYP2C19, CYP2D6
N-desmethylfluoxetine	207	3.8	CYP2C9, CYP2C19, CYP2D6
**Quetiapine**	78	2.93	CYP3A4
Sertraline	91	5.06	CYP2B6, CYP2C19
**Olanzapine**	72	3.61	UGT1A4, CYP1A2
**Mirtazapine**	62	2.9	(CYP3A4, CYP1A2, CYP2D6)
**(Es)**citalopram	49(Es)/32	3.58	CYP2C19
**Risperidone**	27	3.27	CYP2D6
**9OH-risperidone**	27	2.3	Renal excretion
